# Radionuclide theranostics: advances and clinical applications in lung cancer

**DOI:** 10.3389/fonc.2026.1782938

**Published:** 2026-04-13

**Authors:** Yang Yang, Jiang Fu, ShengJie Tang, Tao Liu, HaiYang Hu, HaiYang Guo, Long Wen, YunLong Yang, ChengKuan Liu, YiXin Zhang, Li Yu, HaiNing Zhou

**Affiliations:** 1 Department of Thoracic Surgery, Suining Central Hospital, Suining, Sichuan, China; 2 Institute of Surgery, Graduate School, North Sichuan Medical College, Nanchong, China; 3 Institute of Surgery, Graduate School, Chengdu University of Traditional Chinese Medicine, Chengdu, China; 4 Department of Thoracic Surgery, Affiliated Hospital of Southwest Medical University, Luzhou, China; 5 Department of Anesthesiology, Yongchuan Hospital, Chongqing Medical University, Chongqing, China; 6 Department of Physical Examination, Suining Central Hospital, Suining, Sichuan, China

**Keywords:** molecular imaging, radionuclide therapy, radiopharmaceuticals, targeted therapy, theranostics

## Abstract

Lung cancer remains a leading cause of global cancer deaths, underscoring the urgent need for more accurate diagnostic and treatment approaches. Radionuclide theranostics has rapidly emerged as a paradigm that couples molecular imaging with targeted internal irradiation, enabling personalized cancer care. This review provides a systematic overview of radionuclide theranostics in lung cancer. We first summarize core theranostic principles, including the classes of diagnostic and therapeutic radionuclides and their mechanisms of action. We then emphasize target selection, discussing key molecular targets overexpressed in lung tumors and within the tumor microenvironment that are suitable for radiopharmaceutical development. We synthesize clinical translational evidence by collating efficacy and safety outcomes from pivotal studies and highlighting candidate agents currently in clinical trials. We also examine patient stratification, combination strategies, and the management of treatment-related toxicities. Finally, we outline major challenges, such as target heterogeneity, uncertainties in optimal dosing, and incomplete toxicity profiling, and propose future directions to optimize ligand design, expand target and radionuclide repertoires, and integrate radionuclide therapy with other treatment modalities.

## 1 Introduction

Accounting for about 12.4% of all cancers, lung cancer ranks among the most common malignancies globally and is still the top cause of cancer-related death, with persistent challenges in clinical diagnosis and treatment (1) (2). Pathologically, it is categorized into non–small cell lung cancer (NSCLC), small cell lung cancer (SCLC), and pulmonary neuroendocrine neoplasms (NENs) (3). NSCLC constitutes about 85% of cases, with common variants including adenocarcinoma and large-cell carcinoma, whereas SCLC comprises approximately 15%,and the staging divides cases into limited disease and extensive disease (4, 5). Despite the adoption of a comprehensive, imaging- and biopsy-based approach integrating surgery, chemotherapy, targeted therapy, and immunotherapy, overall survival in most countries remains approximately 10%–20% (6). To address the limitations of current treatments, precision medicine is gaining prominence. As an emerging strategy, radionuclide theranostics leverages molecular targeting to offer substantial clinical potential in lung cancer (7).

Radionuclide theranostics is conceptually linked to radionuclide therapy; however, its hallmark is the pairing of radionuclide-based radiopharmaceuticals to enable molecular imaging and deliver targeted tumor therapy, either independently or in combination (8). Mechanistically, the radionuclide is coupled via a spacer to a binding moiety—such as a peptide, antibody, or small-molecule ligand—to form a targeted complex that binds selectively to cell-surface receptors or antigens on tumor cells, thereby enabling lesion localization for diagnosis and precise therapy (9). The clinical adoption of this concept in nuclear medicine dates back decades. In 1946, radioactive iodine was introduced for both the diagnosis and treatment of thyroid cancer, establishing an early paradigm (10) and laying the groundwork for radionuclide-based oncology, which continued to evolve over subsequent decades (11). More recently, Somatostatin Receptor 2(SSTR2)-targeted radionuclide therapy has substantially improved disease control in neuroendocrine tumors (NETs), and Prostate-specific membrane antigen(PSMA)-targeted radionuclide therapy has enhanced outcomes in prostate cancer, particularly in advanced disease; both have successfully translated from the bench to the bedside (12, 13). Nonetheless, critical constraints remain: the catalog of high-affinity radioligands and validated targets is limited, encompassing only a subset of tumor types, and is insufficient to meet diverse clinical needs. Encouraging signals have emerged in thoracic malignancies: [^177^Lu]Lu-DOTATATE, which targets SSTR2, has shown favorable efficacy in pulmonary neuroendocrine tumors (pulmonary NETs) (14, 15), while [^177^Lu]Lu-FAP-2286, which targets fibroblast activation protein (FAP), has demonstrated notable tumor-suppressive activity in lung squamous cell carcinoma (16). Collectively, ongoing research and clinical practice are converging on the development of next-generation radionuclide therapy agents that couple a favorable safety profile with robust antitumor efficacy. As these efforts advance, radionuclide theranostics is poised to provide improved diagnostic and therapeutic options for a broader range of cancers, including lung cancer (LC), thereby supporting more precise and efficient clinical management.

This review synthesizes the landscape of radionuclide technologies in lung cancer, spanning clinical applications and recent advances. We delineate radionuclide classes and physicochemical properties (e.g., half-life, radiation type), analyze the radiopharmaceutical “radionuclide–carrier” architecture and matching principles to establish a theoretical framework, summarize molecular targets and corresponding radioactive imaging agents in lung cancer, and outline therapeutic pathways of radionuclide-based interventions, including eligible populations and pivotal efficacy evidence. Building on this foundation, we examine practical constraints in radionuclide theranostics with emphasis on target specificity, toxicity management, resistance mechanisms, and radiation safety, and discuss the attendant risks to inform clinical translation.

## 2 Common radiopharmaceuticals and mechanistic principles

**Figure 1:**
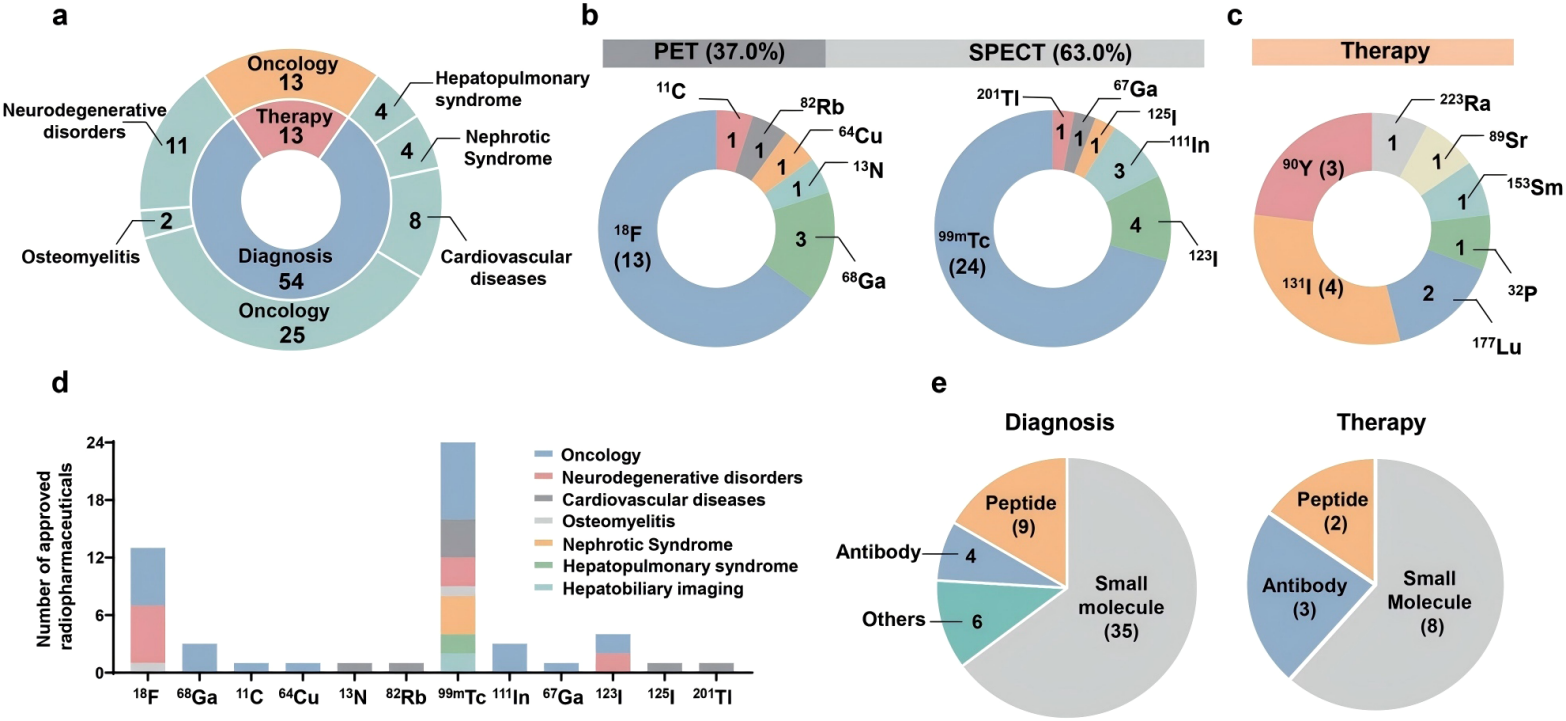
Summary of approved radiopharmaceuticals. **(a)** Approval radiopharmaceuticals used in diagnosis and therapy for different diseases.Diagnostic agents are categorized into seven categories on the basis of indications. Several agents are used in multiple-diseases ([^18^F]FDG, for example), and they are preferentially categorized into their primary indications. All therapeutic radiopharmaceuticals are applied for oncology. **(b)** Radionuclides used in PET (37.0%) and SPECT (63.0%) scanning. Fluorine-18, gallium-68, carbon-11, nitrogen-13, copper-64, and rubidium82 labelled agents are approved for PET/CT diagnosis. For SPECT/CT imaging, technetium-99m, iodine-123, indium-111, gallium-67, iodine125, and thallium-201 are used. **(c)** Radionuclides used in cancer therapy, including iodine-131, yttrium-90, lutetium-177, phosphorus-32, strontium-89, samarium-153, and radium-223. **(d)** Numbers of approved diagnostic radiopharmaceuticals used in various diseases catalogued by radionuclides. Technetium-99m is mostly used in clinical imaging for multiple diseases. Fluorine-18 is used mainly in oncology and neurodegenerative disorders. **(e)** Targeting vectors for diagnostic and therapeutic radiopharmaceuticals. Small molecules are used as the major vectors for radiopharmaceuticals discovery. Peptides play a distinct role both in diagnosis and therapy, particularly after the FDA approval of [^68^Ga]/[^177^Lu]Ga-DOTA-TATE for NETs. Antibodies play essential roles in both imaging and therapy because of their strong binding affinity *in vivo*. Others indicate protein and serum albumin-based radiopharmaceuticals. The number of approved radiopharmaceuticals in each catalogue is presented. *Copyright ^©^ 2025*, The Siqi Zhang, Xingkai Wang, Xin Gao, Xueyao Chen, Linger Li, Guoqing Li, Can Liu, Yuan Miao, Rui Wang, Kuan Hu.

The International Commission on Radiological Protection (ICRP) compendium catalogs roughly 1,200 radionuclides, yet only a few dozen are used clinically or widely in research (17). Because radionuclide therapy can cause adverse events—including primary nephrotoxicity and hematologic toxicity (18)—careful radionuclide selection is critical to maximize efficacy while minimizing toxicity. By clinical purpose, radionuclides are classified as diagnostic or therapeutic (19). Diagnostic radionuclides comprise positron emitters (e.g., ^18^F, ^68^Ga, ^11^C) and gamma emitters (e.g., ^99m^Tc, ^111^In) used for PET and SPECT imaging. Therapeutic radionuclides include alpha emitters (e.g., ^225^Ac, ^212^Pb/^212^Bi, ^211^At, ^223^Ra), beta emitters (e.g., ^177^Lu, ^90^Y, ^131^I, ^67^Cu), and Auger electron emitters (e.g., ^125^I, ^111^In) ^(^20, 21). Some radionuclides emit multiple radiations during decay and can support both imaging and therapy; these “mixed emitters”—such as ^177^Lu, ^131^I, and ^111^In, which also emit gamma rays—enable treatment with synchronous imaging (18, 22) (see **Figure 1**). It should be noted that the γ-rays emitted by such theranostic radionuclides are primarily utilized for post-therapy planar scintigraphy or SPECT to assess *in vivo* biodistribution and facilitate approximate dosimetry. However, the image quality and spatial resolution achieved with these modalities are insufficient to meet the standards of diagnostic PET/CT. Consequently, in clinical practice, precise pre-therapeutic diagnosis typically relies on PET tracers labeled with positron-emitting radionuclides, such as 68Ga or 18F. This establishes the classic theranostic paradigm, wherein a diagnostic PET tracer is paired with a therapeutic radiopharmaceutical (23).

Radiopharmaceutical agents use ionizing emissions from radionuclides to cause DNA damage in cancer cells, producing both single- and double-strand breaks, thereby achieving cytotoxicity. Key modes of action involve lethal effects from direct DNA injury and the generation of reactive oxygen species (ROS)–driven indirect injury and cell death, and immunogenic cell death (9). Radionuclides differ in radiophysical properties, yielding distinct spectra of dominant DNA damage. Beta emitters (e.g., ^177^Lu, ^90^Y) have relatively long ranges and generate a crossfire effect that covers larger lesion volumes, making them suitable for bulky, intrinsically heterogeneous tumors; these agents are used in both preclinical and clinical therapy (18). Their linear energy transfer (LET) is low (approximately 0.1–10 keV/μm), favoring SSBs and minor base modifications, and they elicit indirect DNA damage via ROS-driven oxidative stress (24). Because this indirect effect is oxygen dependent, efficacy may be attenuated in hypoxic tumors (25). Alpha emitters (e.g., ^223^Ra, ^211^At) deliver high energies (approximately 4–8 MeV), short ranges (approximately 28–100 μm), and very high LET (approximately 50–230 keV/μm); At low to intermediate dosing, these agents efficiently cause irreversible DSBs, leading to strong cell-killing activity (26, 27). Alpha particles can also potentiate antigen-specific T-cell responses and act independently of oxygen, conferring potential advantages in hypoxic tumor microenvironments (28). Auger electron emitters (e.g., ^125^I, ^111^In) have ultrashort ranges; although their energies (eV–keV) are lower than those of alpha/beta emitters, microscopic dose localization yields relatively high effective LET (approximately 4–26 keV/μm). When radiolabels localize to the nucleus or near DNA, they efficiently induce DSBs and lethal damage (29). Their short range limits exposure to adjacent normal tissues, making them well-suited for small-volume lesions at sensitive sites (30). Unlike beta particles, Auger electrons predominantly cause direct DNA damage with minimal ROS-mediated indirect effects, reflecting their emission characteristics and nanoscale dose-distribution profile (30). Despite these physical and biological advantages, therapeutic applications remain limited outside a small number of clinical studies (see **Table 1**).

**Table 1:** Characteristics of radionuclides used in radionuclide theranostics.

Radionuclide	Radionuclide half-life	Range in soft tissue (mm)	Mean Eα/β- (MeV)	Indication	Ref
β					
^90^ Y	64.1 h	3.6 mm	1.349 MeV	Liver tumours, primary hepatocellular carcinoma (HCC), and unresectable primary colorectal cancer	(124–128)
^131^ I	8.0 d	0.4 mm	0.356 MeV	Thyroid cancer	(127–130)
^153^ Sm	46.5 h	0.7 mm	–	Cancer that has migrated to the skeleton	(127, 128, 131)
^177^ Lu	6.6 d	0.28 mm	0.208 MeV	Metastatic castration-resistant prostate cancer	(127, 132)
^32^ P	14.26 d	2.6 mm	1.015 MeV	Myeloproliferative neoplasms	(127, 128, 133)
^89^ Sr	50.53 d	2.4 mm	0.908 MeV	Osseous (bony) metastases of bone cancer	(127, 128, 134)
^169^ Er	9.4 d	0.3 mm	–	Synovitis	(127, 128)
^186^ Re	3.72 d	1.2 mm	0.769 MeV	Bone pain palliation, arthritis	(127, 129, 135)
^188^ Re	17 h	2.1 mm	1.592 MeV	Painful bone metastases Medullary carcinoma	(127, 128, 136, 137)
^166^Ho	26.76 h	–	0.628Mev	NETs, colorectal cancer, small hepatocellular carcinoma (SCC)	(127, 138, 139)
α					
^223^ Ra	11.44 d	0.054 mm	6.59 MeV	Bone pain palliation	(127, 140)
^211^ At	7.2 h	0.057 mm	6.79 MeV	RIT for blood cancers like leukemia; brain tumors treated with RIT; RLT for prostate malignancy	(127, 128, 141)
^213^ Bi	46 mins	0.078 mm	8.32MeV	RIT leukemia,	(128, 135)
^225^ Ac	10 d	0.05–0.08 mm	0.218MeV	RLT for prostate cancer	(30, 127)
^227^Th	18.7 days	–	5.9 MeV	Blood and bone marrow cancers	(142)
Electron capture					
^111^In	2.80 days	–	0.245Mev	Oesophageal cancer	(143)

## 3 Molecular imaging targets and diagnostic probes for lung cancer

In the diagnosis of lung cancer, selecting the right molecular target is foundational to clinical efficacy and safety. An ideal target exhibits high specificity and relatively uniform expression in tumor cells or the tumor microenvironment, enabling accurate radioligand delivery and potent tumor cell kill. It should show limited distribution in normal tissues and demonstrate favorable pharmacologic behavior—high internalization efficiency with advantageous membrane trafficking/retention—to provide high-contrast imaging while minimizing normal-tissue radiation. Given the pronounced heterogeneity of lung cancer, identifying targets that balance prevalence, specificity, and druggability remains a central challenge. Under these criteria, target selection ultimately governs the overall performance of lung cancer theranostics (see **Table 2**).

**Table 2:** Overview of targets and properties of tracers for PET/SPECT imaging of lung cancer.

Targets	Location	Tracers	Properties	Classification (cell lines)	Tumor uptake	Study type	Ref
PD-L1	T	[^89^Zr]Zr-atezolizumab	mAbs	NSCLC	Median SUVmax: 9.7	C	(76)
		[^68^Ga]Ga- NOTA-WL12	Peptides	NSCLC	Median SUV_max_: 3.1	C	(144)
		[^18^F]F-BMS-986192	Proteins	NSCLC	Median SUV_max_: 6.5	C	(145)
		[^99m^Tc]Tc-NM-01	sdAbs	NSCLC	TBR: 2.3	C	(146)
		[^89^Zr]Zr-DFO-6E11	mAbs	NSCLC (HCC827)	%ID/g: 5.1 TBR: 12.8	P	(147)
		[^89^Zr]Zr-C4	mAbs	NSCLC (PDX, A549)	%ID/g: 5.0	P	(148)
		[^124^I]I-SIB-SHR-1316	mAbs	NSCLC (PDX)	SUV: ~0.5 TBR: 5.3	P	(149)
		[^64^Cu]Cu-NOTA-Nb6	HCAb	NSCLC (A549)	TBR: 2.1	P	(150)
		[^68^Ga]Ga-NOTA-RW102	Nanobody	NSCLC	SUVmax 5.45	C	(151)
		[^89^Zr]Zr -durvalumab	mAbs	NSCLC	Median SUVmax: 4.9	C	(83)
CCK-2R	T	[^111^In]In-IP-001	Small molecules	NSCLC (A549)	%IA/g: 2.4 TBR: 15.7	P	(152)
		[^68^Ga]Ga-DOTA-MGSS	Small molecules	SCLC	–	C	(153)
EGFR	T	[^18^F]F-IRS	Small molecules	NSCLC	SUV_max_: 2.4 (Mutant)	C	(154)
		[^18^F]F-icotinib	Small molecules	NSCLC (A549)	%ID/g: 0.9	P	(155)
		[^18^F]F-MPG	Small molecules	NSCLC	SUVmax ≥ 2.2 (Mutant) SUVmax < 2.2 (Wild-type)	C	(72)
		[^18^F]F-afatinib	Small molecules	NSCLC	TBR ≥ 6.0 (Mutant) TBR < 6 (Wild-type)	C	(156)
		[^18^F]F-FEA-erlotinib	Small molecules	NSCLC (HCC827)	%ID/g: 0.7 TBR: 1.4	P	(157)
		[^11^C]C-PD153035	Small molecules	NSCLC	SUVmax ≥ 2.9	C	(158)
		[^89^Zr]Zr-cetuximab	mAbs	NSCLC	SUVmax: 1.18—4.74	C	(159)
		[^64^Cu]Cu-NOTA-panitumumab	mAbs	NSCLC	SUVmean 4.70 ± 0.42	P	(160)
c-Met	T	[^89^Zr]Zr-DFO-H2	Diabodies	NSCLC (HCC827, HCC827-GR6)	%ID/g: 1.1 (HCC827) %ID/g: 1.8 (HCC827-GR6)	P	(161)
		[^89^Zr]Zr-PRS-110	Proteins	NSCLC (H441)	%ID/g: 5.9	P	(162)
		[^89^Zr]Zr-Onartuzumab	mAbs	NSCLC (HCC827, HCC827ErlRes)	%ID/g: 30.2 (HCC827) %ID/g: 38.1 (HCC827ErlRes)	P	(163)
		[^68^Ga]Ga-EMP-100	Small molecules	NSCLC	–	C	(164)
		[^18^F]F-FPC	Small molecules	NSCLC (H1993)	%ID/g: 2.5 TBR: 2.4	P	(165)
c-Met & EGFR	T	[^89^Zr]Zr-DFO-amivantamab	Bispecificantibody	NSCLC (HCC827)	*In vitro*	P	(166)
FRα	T	[^18^F]F-AzaFol	Small molecules	NSCLC	%IA/g: 0.5	C	(167)
NTR	T	[^18^F]F-NT	Small molecules	NSCLC (H1299)	%ID/g: 1.9 TBR: 7.8	P	(168)
SSTR_2_	T	[^99m^Tc]Tc-depreotide	Peptides	Lung carcinoids NSCLC	TBR: 2.6	C	(169)
		[^68^Ga]Ga-DOTATOC (octreotide)	Peptides	NSCLC	Mean SUV: 2.0	C	(170)
		[^68^Ga]Ga-DOTATATE (octreotate)	Peptides	SCLC (NCI-H69)	SUV_max_: > 20 (positive) TBR: 12.6	CP	(171)
		[^68^Ga]Ga-DOTA-PA1	Peptides	NSCLC (A549)	%ID/g: 5.2	P	(172)
		([^124^I]I, Mn) OCT-PEG-MNPs	Nanoparticles	SCLC (NCI-H69)	%ID/g: 8.0	P	(173)
		[^68^Ga]Ga-SSO-120	Peptides	SCLC	SUV_max_: > 11.2 ± 8.8	C	(174)
CD 146	T	[^64^Cu]Cu-NOTA-YY146	mAbs	NSCLC (H460)	%ID/g: 7.4	P	(175)
CEA	T	[^99m^Tc]Tc-anti-CEA nanobody	Nanobody	NSCLC (H460)	%ID/g: ~3.0	P	(176)
CXCR4	T	[^68^Ga]Ga-Pentixafor ([^68^Ga]Ga-CPCR4-2)	Peptides	NSCLC SCLC	Average SUV_max_: ~8.5 Average SUV_max_: ~12.0	C	(177)
		[^64^Cu]Cu-AMD3100	Small molecules	LC (3LL: CXCR4-transfected)	%ID/g: 12.3	P	(178)
		[^18^F]F-AlF-NOTA-QHY-04	Peptides	SCLC	%ID/mL 4.98 ± 0.98	P	(67)
		[^89^Zr]Zr-MDX-1338	mAbs	NSCLC (H1155)	%ID/g: 36.2 TBR: 41.0	P	(179)
VEGFR-2	T	[^64^Cu]Cu-NOTA-RamAb	mAbs	NSCLC (HCC4006)	%ID/g: 9.4 ± 0.5	P	(180)
CD30	T	[^89^Zr]Zr-Df-BV	mAbs	NSCLC (H460)	%ID/g: 9.9	P	(181)
CD38	T	[^89^Zr]Zr-Df-daratumumab	mAbs	NSCLC (A549)	%ID/g: 8.1	P	(182)
CD133	T	[^89^Zr]Zr-DFO-αCD133	mAbs	SCLC (NCI-H82)	%ID/g: 50.8	P	(183)
Integrin α_v_β_6_	T	[^18^F]F-α_v_β_6_-BP	Peptides	NSCLC	SUV_max_: 1.0–13.5 TBR: 17.3–67.5	C	(184)
		[^68^Ga]Ga-SFITGv6	Peptides	NSCLC	Mean SUV_max_: 3.3	C	(185)
		[^68^Ga]Ga-DOTA-R01-MG	Peptides	NSCLC (H1975)	%ID/g: 2.5 TBR: 5.2	P	(186)
		[^68^Ga]Ga-avebehexin	Peptides	NSCLC (H2009)	%ID/g: 0.6 TBR: 10.8	P	(187)
Integrin α_v_β_3_	T/TME	[^18^F]F-galacto-RGD	Peptides	NSCLC	Mean SUV: 2.7	C	(188)
		[^18^F]F-AlF-NOTA-PRGD2 (^18^F-Alfatide I)	Peptides	NSCLC (CMT-167)	Mean SUV: 2.9 TBR: 5.9	C	(189)
		[^68^Ga]Ga-DOTA-E-(cRGDfK)2	Peptides	NSCLC	Median SUV_max_: 4.3	C	(190)
		[^68^Ga]Ga-NODAGA-THERA-NOST	Peptides	Lung carcinoids	SUV_max_: 4.8 TBR: 1.5	C	(191)
		[^99m^Tc]Tc-RGD-4CK	Peptides	NSCLC	%ID/g: 4.1 TBR: 3.8	P	(192)
Integrin α_2_β_1_	T/TME	[^68^Ga]Ga-DOTA-A2B1	Peptides	NSCLC (A549)	%ID/g: 2.5 TBR: 1.5	P	(193)
SSTR_2_ & Integrin α_v_β_3_	T/TME	[^68^Ga]Ga-NOTA-3P-TATE-RGD	Peptides	NSCLC SCLC Lung carcinoids	Mean SUV_max_: 4.1 Mean TBR: 5.2 TBR: 4.5 (NSCLC) 6.1 (SCLC) 36.1 (Lung carcinoids)	C	(194)
FAP& Integrin α_v_β_3_	T/TME	[^68^Ga]Ga -FAPI-RGD	Peptides	NSCLC	SUVmax 6.9 ± 5.3	C	(101)
NRP-2	T/TME	[^131^I]I-anti-NRP-2	mAbs	NSCLC (A549)	%ID/g: 4.6 TBR: 3.8	P	(195)
CTLA-4	T/TME	[^64^Cu]Cu-DOTA-ipilimumab	mAbs	NSCLC (A549)	%ID/g: 9.8	P	(196)
FAP	TME	[^18^F]F-FAPI-74 [^68^Ga]Ga-FAPI-74	Small molecules	NSCLC	Average SUV_max_: 12.7 Average SUV_max_: 11.4	C	(197)
		[^68^Ga]Ga-FAPI-04	Small molecules	LC	Average SUV_max_:> 12	C	(198)
		[^68^Ga]Ga-FAP-2286	Peptides	NSCLC	SUV_max_: 7.3	C	(16)
		[^68^Ga]Ga-FAPI-46	Small molecules	NSCLC	SUVpeak 10.3,	C	(199)
PD-1	TME	[^89^Zr]Zr-nivolumab	mAbs	NSCLC	Median SUV_max_: 6.4	C	(145)
		[^89^Zr]Zr-pembrolizumab	mAbs	NSCLC	Mean SUV_max_: 6.5	C	(200)
TIGIT	TME	[^68^Ga]Ga-GP12	Peptides	NSCLC	SUV_max_: 4.8	C	(201)

### 3.1 FAP

Fibroblast activation protein (FAP) functions as a discriminating signature in the tumor milieu and shows strong overexpression in fibroblasts associated with pulmonary malignancies (31), whereas most normal adult tissues show little to no detectable expression. The enrichment of FAP within neoplastic tissue, coupled with minimal presence in healthy organs, makes it well-suited for imaging and for determining disease stage. In lung cancer and numerous other tumors, higher FAP levels are linked to worse outcomes (32), underscoring its translational relevance. Leveraging FAP specificity, FAP inhibitor–based compounds have been engineered as radiotracers. Among these, the ^68^Ga-FAPI series (e.g., FAPI-02, FAPI-04, FAPI-46) is well suited to PET imaging and has become a mainstay in ongoing clinical research and applications.

Uptake on FAPI PET/CT shows a strong correlation with FAP levels in tissue and has been confirmed across multiple multicenter studies. In 141 patients spanning 14 solid tumor types, Mona and colleagues documented a robust positive correlation between [^68^Ga]Ga-FAPI-46 SUVmax and IHC-derived FAP scores (33). In an LC cohort, researchers led by Wei reported a highly significant association. Their analysis of six resected tissue samples demonstrated that the intensity of [^18^F]F-FAPI-04 signal closely corresponded with levels of fibroblast activation protein confirmed by immunohistochemistry (34). These data support the use of FAPI PET/CT uptake as a noninvasive surrogate for evaluating the prognostic relevance of FAP expression. For lesion detection, FAPI imaging demonstrates advantages in LC. Chen showed widespread FAP expression in lung tumors, with FAP positivity of 100% in squamous cell carcinoma and 85.7% in adenocarcinoma. Among 12 patients with early-stage adenocarcinoma, only 3 were positive on [^18^F]FDG PET/CT, whereas IHC revealed FAP positivity in 10, suggesting potential utility of [^68^Ga]Ga-FAPI PET/CT for early disease detection (35). In 27 primary or recurrent lung cancer lesions, Wang and co-authors reported a higher mean SUVmax for [^68^Ga]Ga-FAPI compared with [^18^F]FDG (13.7 vs 10.4), accompanied by an increased TBR (34.2 vs 25.9; p=0.02) (36). In patients with locally advanced lung adenocarcinoma, Loktev and colleagues found that [^68^Ga]Ga-FAPI-02 had greater metastatic uptake and a lower background signal than [^18^F]FDG, yielding superior image contrast and sharper primary tumor margins (37). In selected clinical scenarios, the incremental benefit of FAPI imaging is more evident: A case was detailed by Giesel’s team where [^18^F]FDG FDG PET/CT detected only a small T2a primary lesion and suggested N2 nodal involvement, with no evidence of distant metastasis, while [^68^Ga]Ga-FAPI-04 PET/CT detected two intracranial lesions later confirmed as brain metastases on contrast-enhanced MRI (38). Another study aimed to evaluate the diagnostic performance of [^68^Ga]Ga-FAPI-04 PET/CT in detecting lymph node metastases in non-small cell lung cancer (NSCLC) and to investigate the correlation between tumoral [^68^Ga]Ga-FAPI-04 uptake and fibroblast activation protein (FAP) expression. This retrospective study enrolled 91 patients with NSCLC, using pathological findings or typical imaging features as the reference standard. The results demonstrated that [^68^Ga]Ga-FAPI-04 PET/CT achieved a sensitivity and positive predictive value of 96.70% and 100%, respectively, for the detection of primary tumors. For lymph node metastasis detection, the sensitivity, specificity, and accuracy were 72.00%, 93.10%, and 89.36%, respectively. This modality demonstrated superior performance compared to conventional [¹^8^F]FDG PET/CT, which is prone to interference from inflammatory processes, whereas the high specificity of [^68^Ga]Ga-FAPI-04 provides valuable support for precise clinical staging and treatment decision-making. False-negative findings were predominantly observed in small metastatic lesions with a short-axis diameter of <0.5 cm, suggesting that the detection capability of [^68^Ga]Ga-FAPI-04 for small or low-activity metastases remains constrained by the spatial resolution of PET (39). Similarly, Liu et al. reported that for mediastinal-type LC, [68Ga]Ga-FAPI PET/CT clearly delineated mediastinal disease, avoiding lesion obscuration from physiologically high myocardial uptake on [^18^F]FDG PET/CT (40). Collectively, available evidence indicates that [^68^Ga]Ga-FAPI performs comparably to, and often better than, [^18^F]FDG used to identify both primary tumors and metastatic sites.

FAPI PET/CT offers several advantages—greater target specificity, low background, rapid acquisition, and a relatively low radiation dose (41). It can also complement [^18^F]FDG to refine tumor delineation for surgical and radiotherapy planning (42–45). Future efforts may focus on integrating high-sensitivity detection technologies or developing novel tracer derivatives to further enhance the identification of early micro metastases.

### 3.2 SSTR2

Somatostatin receptors belong to the GPCR family and are broadly present in many normal tissues as well as diverse tumors. Five subtypes have been identified (SSTR1–SSTR5) (46). In pulmonary neuroendocrine neoplasms (NENs; e.g., pulmonary carcinoids) and thymic neuroendocrine tumors, SSTRs are typically highly expressed. Notably, the SSTR expression profile of pulmonary carcinoids closely resembles that of gastroenteropancreatic neuroendocrine tumors (GEP-NETs) (47).

Building on the high expression of SSTRs in NENs, SSTR-targeted molecular imaging has become a key tool. [^68^Ga]Ga-DOTATATE binds specifically to SSTRs to enable targeted imaging, thereby compensating for the limited detection sensitivity of [^18^F]FDG in NENs. Many pulmonary carcinoids with good differentiation fail to demonstrate elevated FDG uptake, whereas [^68^Ga]Ga -DOTATATE provides effective visualization (48). In routine NEN imaging, [^68^Ga]Ga -DOTATATE has largely replaced [^111^In]In pentetreotide as the preferred modality for typical (well-differentiated) pulmonary carcinoids (49). Its value in staging is particularly notable, and it reveals tiny metastatic deposits that conventional imaging often misses and aids in identifying candidates for peptide receptor radionuclide therapy (PRRT). Accordingly, both the NCCN(National Comprehensive Cancer Network) and NANETS(North American Neuroendocrine Tumor Society) recommend SSTR PET imaging at initial staging for all patients with NENs (50). Within this framework, a team at University Hospital Essen further explored the potential of SSTR imaging in more aggressive small-cell lung cancer (SCLC). In a retrospective study of 54 newly diagnosed SCLC patients, they performed imaging with a novel SSTR2-antagonist PET tracer, [^68^Ga]Ga SSO120. Uptake of [^68^Ga]Ga SSO120 correlated strongly with SSTR2 immunohistochemical expression in tumor tissue, indicating that this technique can reliably and noninvasively assess target expression. Notably, more than 75% of patients showed tumor uptake above the hepatic background, providing critical support for applying SSTR2-targeted radionuclide therapy in SCLC (51). These findings not only support the diagnostic potential of [^68^Ga]Ga SSO120 PET in aggressive lung cancers but also lay a translational foundation for extending the NEN theranostic paradigm to SCLC.

SSTR2-targeted PET imaging has notable limitations. Diagnostic performance varies by histopathologic subtype; in more aggressive entities, such as atypical pulmonary carcinoids, [^18^F]F-FDG frequently outperforms SSTR tracers (e.g., [^68^Ga]Ga -DOTATATE) (52). Using both modalities can provide complementary information for staging and for evaluating treatment response. However, the clinical utility of this strategy is largely confined to SSTR-positive pulmonary and thymic neuroendocrine neoplasms. Its diagnostic yield for other lung cancer subtypes, including NSCLC, is limited; thus, application should be tailored to the tumor pathology and the specific clinical scenario.

### 3.3 PSMA

Prostate-specific membrane antigen (PSMA) is a cell−surface glutamate carboxypeptidase that participates in folate−related metabolic processing and transport. Its expression across diverse tumors correlates positively with malignant potential (53). Besides its strong presence in prostate carcinoma cells, PSMA localizes to endothelial cells forming the new vasculature in numerous solid cancers—including lung cancer—with particularly pronounced expression in the tumor neovasculature of high-grade (G3/G4) NSCLC. PSMA expression may also be associated with brain metastasis in LC (54, 55). Within NSCLC, *in vitro* evidence points to PSMA expression rates of 64% for squamous tumors, 71% of large-cell carcinomas, and 45% of adenocarcinomas, most commonly localized to neovascular endothelium (56). In small cell lung cancer (SCLC), approximately 70% of cases exhibit PSMA expression, which is predominantly—if not exclusively—confined to neovascular endothelium (55). Data from preclinical models indicate that PSMA fosters neovascular growth in tumors through matrix breakdown and activation of integrin-dependent signal cascades (57).

Clinically, accumulating reports support the utility of PSMA imaging in lung cancer. Usmani’s team detailed a 73-year-old man with prostate cancer who was evaluated with [^68^Ga]Ga -PSMA PET/CT due to PSA elevation; imaging revealed a focal pulmonary lesion (SUVmax 5.6) that was histologically confirmed as lung adenocarcinoma (58). Furthermore, compared with benign lesions, PSMA uptake is significantly higher in intrapulmonary lesions of primary lung cancer (PLC) and in mediastinal nodal metastases. Relative to fluorodeoxyglucose PET/CT, PSMA PET/CT significantly reduces false-positive findings during lymph node staging. This improved accuracy allowed for correct nodal stage revision in 12 of 20 patients, potentially averting unnecessary chemoradiotherapy. Notably, that study also provided the first pathology-based evidence linking PSMA imaging results in primary lung cancer to tumor neovascularization (59). Lung carcinoma is a leading source of brain spread, with more than half of affected patients developing cerebral metastases over their illness, with poor prognosis. Despite surgery, radiotherapy, and immunotherapy, overall outcomes remain limited, and improving response rates is a key unmet need (60). In this context, [^68^Ga]Ga -PSMA PET/CT shows promise: it clearly demonstrates PSMA-ligand uptake in primary lung tumors and brain metastases, and may serve as a sensitive tool to detect brain metastases and to quantify their number, location, and size. For treatment decision-making, PSMA PET/CT can assess PSMA expression in brain metastases to identify candidates for PSMA radioligand therapy (PSMA RLT) (61).

Despite the diagnostic utility of PSMA-ligand PET/CT, important limitations remain, especially when differentiating pulmonary metastases from prostate cancer from synchronous primary lung cancer. This challenge arises because PSMA uptake is highly heterogeneous across these entities (62), and because PET SUVmax and CT morphologic features do not show meaningful differences. Moreover, the extent of PSMA expression remains contentious. Small histologic studies with limited sample sizes indicate concordant high PSMA expression between primary and metastatic lung lesions; however, this finding does not fully align with PSMA PET imaging and warrants confirmation in larger, prospective cohorts (63).

### 3.4 CXCR4

C-X-C motif chemokine receptor 4 (CXCR4) is a versatile therapeutic and imaging target in oncology. By shaping vascular growth in tumors, controlling invasion/metastatic behavior, and affecting therapeutic resilience, it constitutes a compelling avenue for radionuclide theranostics (64–66). Among CXCR4-directed tracers, Pentixafor currently appears most promising for lung cancer applications. In lung cancer, small cell lung cancer (SCLC) frequently exhibits elevated CXCR4 expression (SUVmax 7.51 ± 3.01), which is significantly higher than that observed in other solid tumors (P < 0.05) (67). This characteristic holds potential for distinguishing molecular subtypes of SCLC—such as SCLC-N versus neuroendocrine subtypes—and may have implications for prognosis and treatment selection (68). However, the heterogeneity of CXCR4 expression in non-small cell lung cancer (NSCLC), the predominant histological type of lung cancer, also warrants attention.A study involving 94 patients with lung cancer reported that NSCLC accounted for 79.8% of cases (n = 75), including 54 squamous cell carcinomas, 16 adenocarcinomas, and 5 not otherwise specified. All NSCLC subtypes exhibited increased tracer uptake on [^68^Ga]Ga-Pentixafor PET/CT, indicating positive CXCR4 expression, with adenocarcinomas demonstrating significantly higher SUVmax values than squamous cell carcinomas. Correlation analysis revealed a significant positive correlation between SUVmax and mean fluorescence intensity (MFI) in both squamous cell carcinoma (r = 0.690) and adenocarcinoma (r = 0.538), confirming the specific binding of the tracer to CXCR4. Using a SUVmax threshold of 6.7, the sensitivity and specificity for distinguishing adenocarcinoma from squamous cell carcinoma were 87.5% and 71.4%, respectively (69).

Notably, the heterogeneity of CXCR4 expression among NSCLC subtypes carries important clinical implications. The higher SUVmax observed in adenocarcinomas suggests that these patients may be more suitable candidates for CXCR4-targeted therapies, which aligns with the biological propensity of adenocarcinomas for early hematogenous metastasis. In contrast, the stronger correlation between SUVmax and MFI in squamous cell carcinomas indicates greater concordance between imaging and pathological findings, potentially attributable to distinct histological features or membrane receptor distribution patterns. These findings provide an imaging-based foundation for identifying patients eligible for targeted radioligand therapies such as [¹^77^Lu]Lu-Pentixather.

Overall, [^68^Ga]Ga-Pentixafor PET/CT is not consistently superior to [^18^F]FDG PET/CT in diagnostic performance, but within precision-medicine frameworks—particularly for CXCR4-based targeted therapy—it offers substantial clinical value and strong future potential (70).

### 3.5 EGFR

The epidermal growth factor receptor (EGFR) belongs to the ErbB receptor family, which includes HER1 (ERBB1), HER2 (ERBB2), HER3 (ERBB3), and HER4 (ERBB4). EGFR is broadly expressed on the surface of epithelial cells, fibroblasts, glial cells, and keratinocytes, and it is required for NSCLC cell growth and viability. Exon 19 deletion and the L858R point mutation are prototypical activating changes that fuel tumor growth, establishing EGFR as a pivotal target for tyrosine-kinase inhibitor treatment and imaging at the molecular level (71).

In NSCLC, EGFR is overexpressed in more than 60% of patients. Among people of Asian ancestry, the rate of EGFR-activating alterations is about 30% to 50%. Preclinical and clinical evidence show that EGFR-targeted PET/CT enables noninvasive assessment of EGFR mutation status, identification of potential TKI responders, monitoring of acquired resistance, and prediction of treatment benefit, thereby informing individualized therapeutic strategies (72). Among EGFR-targeted agents, radiotracers built on tyrosine kinase inhibitor (TKI) scaffolds are particularly promising. For instance, although directly labeled [^11^C]C gefitinib is an EGFR TKI, its uptake in mouse models does not align with tumor EGFR expression—likely due to high lipophilicity leading to nonspecific binding—whereas structurally optimized TKI-derived tracers demonstrate superior imaging performance (73). In a cohort of 75 patients with NSCLC, [^18^F]F MPG PET/CT identified tumors harboring activating EGFR mutations with high sensitivity and specificity, as confirmed by histopathology; moreover, when [^18^F]F MPG SUVmax ≥ 2.23, the EGFR-TKI response rate was approximately twice that of an unselected population, supporting its role as a predictive biomarker of TKI efficacy. Relative to conventional [^18^F]FDG PET/CT, EGFR TKI PET imaging offers distinct advantages. [^18^F]FDG reflects glycolytic activity rather than a specific driver genotype. [^11^C]C erlotinib can detect EGFR-positive lesions in non-enlarged lymph nodes that are [^18^F]FDG-negative in some cases, underscoring its potential to assess tumor heterogeneity and micrometastases (74, 75). Thus, although EGFR TKI PET/CT may be less sensitive than [^18^F]FDG for overall lesion detection, it provides unique value within precision oncology, particularly for guiding EGFR TKI–based therapy, with strong prospects for clinical integration. Collectively, the development and clinical translation of EGFR-targeted radiotracers (e.g., [^18^F]F MPG, [^11^C]C erlotinib) are advancing precision care in NSCLC. With continued optimization and broader clinical deployment in routine NSCLC management, PET tracers targeting EGFR TKIs are set to play a central role.

### 3.6 PD-L1/PD-1

Programmed death-ligand 1 (PD-L1) is pivotal in antitumor immunity, and its level of expression is tightly linked to how well immune checkpoint inhibitors work, therefore PD-L1 is considered a clinically promising biomarker to guide theranostics (76). Immuno−PET, leveraging labeled mAbs and fragment derivatives, allows on−body visualization and measurement of targets such as PD−L1, supporting patient stratification and enabling prediction of therapeutic outcomes (77).

In this context, radiolabeled single-domain antibodies for immuno-PET have advanced rapidly (78). A range of PD-L1–targeted tracers has emerged; notably, the development of a [^68^Ga]Ga-labeled nanobody tracer began with the screening and characterization of high-quality candidates. Through an immunization strategy, investigators generated the high-affinity PD-L1 nanobody RW102 and a derivative, ABDRW102, fused to an albumin-binding domain. Surface plasmon resonance (SPR) confirmed that RW102 and ABDRW102 display favorable binding kinetics to recombinant human PD-L1, supporting their use as precision imaging agents (79, 80). Multiple classes of PD-L1 imaging ligands have progressed through preclinical and clinical evaluation. Li et al. reported [^89^Zr]Zr-DF-KN035, which showed robust diagnostic performance in a glioma xenograft model and yielded interpretable biodistribution in nonhuman primates. In a follow−up trial, researchers assessed imaging tolerability and whole−body distribution in patients whose solid malignancies were PD−L1 positive (81, 82). Radiolabeled durvalumab has also been used for PD-L1 PET/CT in advanced non–small cell lung cancer (NSCLC) (83), and the feasibility of whole-body PD-1/PD-L1 PET in NSCLC has been investigated. The [^68^Ga]Ga-labeled Adnectin [^68^Ga]Ga-BMS-986192 has been synthesized and evaluated preclinically as a PD-L1 imaging agent (84). Peptide-based PET has enabled quantitative assessment of PD-L1 therapeutic target occupancy/engagement (85). PD-L1-binding peptide tracers have completed first-in-human evaluation in NSCLC (86), and an ^18^F-tagged D−peptide that blocks PD−L1 has finished preclinical evaluation and has also been tested in an initial human trial (87). In addition, the anti–PD-L1 Probody immuno-PET tracer [^89^Zr]Zr-CX-072 has undergone first-in-human evaluation, providing preliminary biodistribution and pharmacokinetic data (88). Collectively, PD-L1–targeted immuno-PET shows strong potential in precision oncology, particularly for patient stratification, prediction of therapeutic response, and monitoring during PD-1/PD-L1–directed treatment.

**Figure 2:**
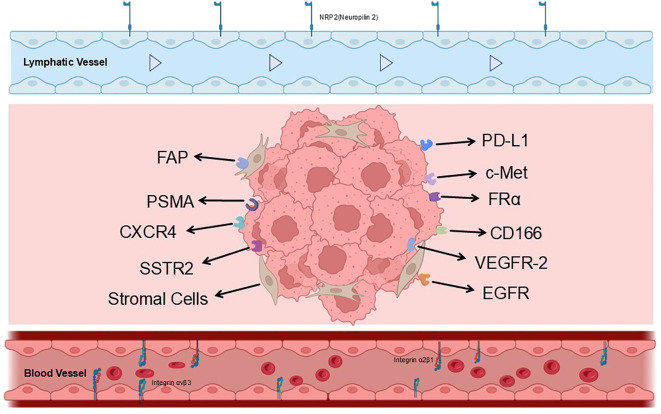
Schematic representation of cellular and molecular architecture in the lung tumor microenvironment for theranostic targeting. This diagram illustrates the spatial distribution of key molecular targets across distinct cellular compartments within a pulmonary tumor, relevant for positron emission tomography (PET)/single-photon emission computed tomography (SPECT) imaging and radionuclide therapy. Abbreviations: NRP2, Neuropilin 2; PSMA, Prostate-specific membrane antigen; VEGFR-2, Vascular endothelial growth factor receptor-2; PD-L1, Programmed death-ligand 1; c-Met, Hepatocyte growth factor receptor; FAP, Fibroblast activation protein; CD166, Activated leukocyte cell adhesion molecule; EGFR, Epidermal growth factor receptor; FRα, Folate receptor alpha; CXCR4, C-X-C chemokine receptor type 4; SSTR2, Somatostatin receptor subtype 2.

Following a synthesis of key targets—including FAP, SSTR2, CXCR4, PSMA, EGFR, and PD-1/PD-L1—it is worth underscoring that lung cancer’s molecular heterogeneity and the complexity of its tumor microenvironment jointly drive the continued expansion of the molecular imaging target landscape (**Figure 2**). The figure outlines the interplay among cancer cell proliferation, immune cells, and stromal components within the tumor, and collates multiple classes of critical membrane proteins, including those emphasized in this review. Beyond targets already in clinical use or under clinical investigation, several emerging targets warrant attention. c-Met, a pivotal signaling axis in tumor invasion and metastasis, shows promise for c-Met–targeted PET to identify NSCLC with MET alterations. A variety of molecular probes—including antibodies, peptides, and small molecules, such as [^64^Cu]Cu-NOTA-rh-HGF, ^89^Zr-labeled probes, and ¹^8^F-labeled probes—have been investigated for c-Met-targeted PET imaging. As a representative example, the novel PET probe [^68^Ga]Ga-NOTA-SL1 demonstrated favorable tumor uptake and targeting performance in non-small cell lung cancer (NSCLC) models, including HCC827 and PC-9 cell lines. Specifically, in the HCC827 model, the tumor uptake reached 2.93 ± 0.64% ID/g at 30 minutes post-injection. This probe also exhibited excellent *in vitro* stability and hydrophilicity, suggesting its potential clinical utility for assessing c-Met expression levels in the diagnosis of lung cancer (89). VEGFR2 is a key receptor involved in tumor angiogenesis. Previous studies have employed ^64^Cu-labeled ramucirumab—a humanized anti-VEGFR2 monoclonal antibody—for PET imaging and confirmed its specific targeting capability in a mouse model of non-small cell lung cancer (NSCLC). These findings highlight the potential of VEGFR2-targeted strategies for both imaging and therapeutic applications in lung cancer (90). Integrin αvβ3 is highly expressed in neovasculature and at invasive fronts, positioning it as a potential imaging biomarker for assessing lung cancer invasiveness and angiogenic activity. Technetium-99m-labeled RGD peptides, such as [^99m^Tc]Tc-3PRGD2, have demonstrated high sensitivity in detecting both primary and metastatic lesions, underscoring their promise as tools for molecular subtyping and biological characterization of lung cancer. However, their translation into therapeutic strategies remains challenging. The expression of integrin αvβ3 in certain benign lesions may compromise imaging specificity, and further research is needed to develop these agents into effective therapeutics (91). Moreover, Chomet et al. employed ^89^Zr-labeled CX-2009, an anti-CD166 antibody–drug conjugate, to perform immuno-PET imaging in a CD166-positive lung cancer mouse model. The results demonstrated high specific uptake of [^89^Zr]Zr-CX-2009 in tumor tissues (21.8 ± 2.3% ID/g), with its distribution closely correlating with CD166 expression levels. The study also revealed that CX-2009 requires proteolytic activation by proteases within the tumor microenvironment before binding to CD166; however, this activation process does not compromise its targeting efficiency. In conclusion, CD166 exhibits favorable expression specificity and targeting potential in lung cancer tissues (92). Overall, most of these emerging targets remain in preclinical or early clinical development, yet collectively delineate a diversified frontier in precision molecular imaging of lung cancer. Looking forward, as more highly specific probes are validated and translated to the clinic, multi-target, multidimensional PET imaging is poised to more comprehensively portray the molecular landscape of LC and to enable more refined individualized treatment decisions.

## 4 Targeted radionuclide therapy for lung cancer

Radionuclide therapy is a targeted radiotherapeutic approach in which radionuclide-labeled ligands specifically bind tumor-associated molecules to deliver precise intralesional irradiation. Success hinges chiefly on high binding affinity to the target and sustained residence within the tumor, aiming to maximize cancer cell kill while minimizing dose to normal tissues. In lung cancer, somatostatin receptor 2 (SSTR2) and fibroblast activation protein (FAP) currently have the strongest preclinical and clinical support for radionuclide therapy. Notably, beyond SSTR2 and FAP, multiple radionuclide therapy agents directed at other targets are being actively explored, each with distinct molecular characteristics and potential applications (see **Table 3**).

**Table 3:** Comparative profile of radionuclide therapy candidates assessed in LC therapy, outlining strengths and limitations.

Radionuclide	Therapeutic compound	Target	Characteristics	Benefits	Limitations	Studies	Type	Ref
^225^Ac α, β, γ	[^225^Ac]Ac-DOTATATE	SSTR_2_	Peptides	Inhibit tumor expansion Comparatively higher effectiveness with reduced adverse effects	Kidney injury Long−term worsening renal disease	Lungcarcinoids (H727, H69	P	(202)
	[^225^Ac]Ac-SC16.56/N149	DLL3	mAbs	Tumor growth suppression Extended life expectancy	Reduced payload−to−antibody loading ratio	SCLC (PDX)	P	(203)
	[^225^Ac]Ac -EBTATE	SSTR2	Peptides	100% survival; 80% complete remission in NCI-H524;	Shrinking of the spleen’s lymphoid (white−pulp) regions; Fatty changes/ballooning degeneration in liver	SCLC (NCI-H524)	P	(117)
^227^Th α, β	[^227^Th]Th-anetumab	MSLN	mAbs	Marked gain in overall longevity *In vivo* effective in a disseminated model of lung cancer	Suppression of white blood cells	NSCLC (NCI-H226)	P	(204)
^131^I β, γ	[^131^I]I-chTNT	Necrotic tumors	mAbs	Tumor necrosis targeted therapy	Bone marrow suppression Hematological toxicities	NSCLC	C	(205)
	[^131^I]I-cNGEGQQc	α_3_β_1_ Integrin	Peptides	Tumor growth suppression	Limited dose administration (High kidney uptake)	NSCLC (H1975, L78)	P	(206)
	[^131^I]I-RGD-BSA-PCL	α_v_β_3_ integrin	Nanoparticles	Extended life expectancy Longer residence times in tumor	Intratumoral injection	NSCLC (NCI-H460)	P	(207)
	[^131^I]I-ERIC1	NCAM1 (CD56)	mAbs	Tumor growth inhibition; Sustained remission; Extended survival	Hematologic toxicity (Leukocyte depletion); Dose-dependent toxicity	SCLC (NCI-H69 xenograft)	P	(208)
	[^131^I]I-TQGMNP	Glucuronidase enzyme	Nanoparticles	Multimodality imaging: SPECT and MRI Tumor growth suppression	Hematologic toxicity	NSCLC (A549)	P	(209)
	[^131^I]I-prohy	Necrotic tumors	Small molecules	Tumor necrosis targeted radiotherapy Tumor growth suppression Extended life expectancy	The precise basis behind protohypericin’s preference for necrotic tissue remains uncertain. Its clinical benefit still requires additional confirmation through further studies.	NSCLC (A549)	P	(210)
^177^Lu β, γ	[^177^Lu]Lu-DOTATATE	SSTR_2_	Peptides	Longer projected lifespan Higher rates of illness stabilization and confirmed tumor shrinkage Longer time without worsening of the condition Reduced risk metrics for mortality and for return of the illness	Nephrotoxicity Anemia Thrombocytopenia Hematologic toxicity Myelodysplastic syndrome Leukemia	Lungcarcinoids	C	(112)
	[^177^Lu]Lu-FAP-2286	FAP	Peptides	Significant decrease in lesion size and SUV_max_ Longer tumor retention	Headache Abdominal pain Anemia Hematologic toxicity	NSCLC	C	(211)
	[^177^Lu]Lu-DOTA-HA100-N	CD44 and CD13	Hyaluronan modified by peptide	Targeting malignant cancers with abundant blood vessels	Low tumor accumulation Easily degradable	NSCLC (NCI-H292)	P	(212)
	[^177^Lu]Lu-DOTA-TOC	SSTR2	Peptides	Increased tumor growth delay and survival when combined with PARPi	–	SCLC (H69, H446)	P	(108)
	[^177^Lu]Lu-SC16.56 [^177^Lu]Lu-N149	DLL3	mAbs	Higher drug–antibody ratio	Less effective than agents tagged with 225Ac	SCLC (PDX)	P	(203)
	[^177^Lu]Lu-DOTA-RS7	EGP-1	mAbs	Tumor growth suppression	Decrease in body mass Blood-related adverse effects	NSCLC (Calu-3)	P	(213)
	[^177^Lu]Lu-DOTA-MGS5	CCK2R	Peptides	High tumour uptake in SCLC lesions; Favourable tumour-to-organ ratios; Low predicted kidney/bone marrow dose	Receptor-mediated uptake in stomach (dose-limiting)	SCLC (ED-SCLC)	C	(214)
	[^177^Lu]Lu-EB-RGD	Integrin α_v_β_3_	Peptides	Inhibition of neoplastic expansion Extended circulatory persistence with increased bloodstream retention Pronounced deposition within malignant tissue Prolonged dwell time at the lesion site	Body weight loss	NSCLC (PDX)	P	(215)
	[^177^Lu]Lu-DOTA-E(cRGDfK)2	Integrin α_v_β_3_	Peptides	Restraint of cancerous expansion Marked build−up within malignant sites Swift clearance through the renal pathway	Potential nephrotoxicity and hematological toxicity	NSCLC (A549)	P	(216)
^90^Y β	[^90^Y]Y-DOTATOC	SSTR_2_	Peptides	Extended life expectancy	Nephrotoxicity Anemia Leukopenia Thrombocytopenia	Lung carcinoids	C	(171)
	[^90^Y]Y-FF-21101	Placental (P)-cadherin	mAbs	Full remission observed in individuals exhibiting elevated P−cadherin levels	Diminished P−cadherin levels in pulmonary carcinoid tumors Reduced lymphocyte count Decreased white blood cell count Lowered platelet count	Lung carcinoids	C	(217)
	[^90^Y]Y-MAb-6	CDH3/P-cadherin	mAbs	Tumor growth suppression and regression	Body weight loss	NSCLC (H1373)	P	(218)
^188^Re β, γ	[^188^Re]Re-P2045	SSTR_2_	Peptides	Short plasma half-life Well tolerated	Lymphopenia No responses	NSCLC	C	(115)
	[^188^Re]Re-cetuximab	EGFR	mAbs	Tumor growth suppression Extended life expectancy	Body weight loss	NSCLC (NCI-H292)	P	(219)
	[^188^Re]Re-bevacizumab	VEGF	mAbs	Tumor growth suppression	No significant tumor regression	NSCLC (A549)	P	(220)
^166^Ho β, γ	[^166^Ho]Ho-IG-cisplatin	Guiding therapeutic agents to neoplastic sites with a magnet placed outside the body	Nanoparticles	Combination of radiotherapy with chemotherapy	Absence of direct ligand−guided aiming	NSCLC (A549)	P	(221)

### 4.1 FAPI-targeted radionuclide therapy

The cancer-specific upregulation of fibroblast activation protein (FAP) within the lung tumor microenvironment, coupled with its minimal expression in healthy tissues (see Section 3.1), establishes this protein as a highly attractive candidate for targeted radionuclide therapy. For radionuclide selection, the principal options for FAP-targeted radionuclide therapy include ^177^Lu (β^-^ emitter; half-life, 6.7 days; moderate tissue penetration), ^90^Y (β^-^ emitter; half-life, 64.1 hours; higher energy and penetration), ^225^Ac (α emitter; high linear energy transfer; potent short-range cytotoxicity), and ^153^Sm (β^-^ emitter; half-life, 46.3 hours; suited to the initial phase of combination regimens). All have shown promise in lung cancer therapy (93–96).

As a novel therapeutic option with encouraging efficacy and a favorable safety profile in advanced lung cancer, FAPI-targeted radionuclide therapy provides a viable option, particularly for those whose disease has progressed after multiple therapeutic regimens. Nevertheless, its long-term effectiveness requires confirmation in larger, prospective clinical trials (97). Furthermore, studies by Assadi (98), Yang (99), Rao et al (16) in lung adenocarcinoma and squamous cell carcinoma cohorts reported SD or PR after treatment with [^177^Lu]Lu-FAPI agents, with no significant adverse events or dose-limiting toxicities. In a dual-radionuclide FAPI radionuclide therapy case involving metastatic pulmonary sarcoma, tracer distribution was favorable and treatment was well tolerated, with disease stability sustained over an eight−month period (96). In addition, the dual-target radiotracer [^68^Ga]Ga-FAPI-RGD, which co-targets FAP and integrin αvβ3, demonstrated higher primary tumor detection rates and greater accuracy for mediastinal nodal staging than FDG PET/CT was performed in a group of 51 individuals evaluated for possible lung malignancy, thereby informing subsequent radionuclide therapy target optimization and patient selection (100, 101). To overcome the rapid tumor washout and short retention of conventional FAPI tracers, FAP-2286—a cyclic peptide–based ligand—was developed to improve *in vivo* pharmacokinetics; its nonradioactive metal complex shows strong, specific binding to FAP. FAP-2286 employs a cyclic peptide as its FAP-binding motif, whereas FAPI compounds are developed based on the small-molecule inhibitor UAMC1110. The cyclic peptide structure confers enhanced conformational rigidity and plasma stability to FAP-2286, thereby improving its binding affinity for FAP. In a prospective study, although radiolabeled FAP-2286 and FAPI-46 exhibited comparable early tumor uptake, their pharmacokinetic profiles diverged significantly after conjugation with the therapeutic radionuclide lutetium-177 (¹^77^Lu) (102). [^177^Lu]Lu-FAP-2286 demonstrated substantially prolonged tumor retention, with 16.4% ID/g remaining at 72 h post-injection, compared to only 1.6% ID/g for [^177^Lu]Lu-FAPI-46 at the same time point. Accordingly, the time-integrated activity coefficient and tumor-absorbed dose of FAP-2286 were 12-fold and 9-fold higher, respectively, than those of FAPI-46. Cellular assays further confirmed that FAP-2286 was retained within endosomal compartments for up to 72 h, whereas FAPI-46 was rapidly cleared. The rapid tumor washout of FAPI compounds limits their therapeutic potential when combined with long-half-life radionuclides such as ^177^Lu. In contrast, FAP-2286 combines sustained tumor retention with favorable renal clearance, positioning it as a more suitable candidate for therapeutic applications (103). In murine models, FAP-2286 exhibited rapid and sustained accumulation within FAP-positive tumors alongside low normal-tissue uptake; [^177^Lu]Lu-FAP-2286 significantly suppressed tumor growth in HEK-FAP xenografts (inhibition 111%–113%; p<0.05), outperforming [^177^Lu]Lu-FAPI-46 (104). In a prospective clinical study by Xie Yang and colleagues, nine patients with advanced lung cancer refractory or resistant to standard therapies received [^177^Lu]Lu-FAP-2286. In terms of dosing, a fixed activity of approximately 200 mCi (7.4 GBq) was administered per cycle, with cycles repeated every eight weeks for a minimum of two cycles, resulting in a cumulative activity ranging from 14.8 to 44.4 GBq. Renal protective measures and hydration were routinely implemented before and after each treatment cycle. According to RECIST 1.1 and PERCIST 1.0 criteria, alongside EORTC QLQ-C30 scores, most participants benefited clinically, experiencing clear improvements in QoL and symptom control (notably dyspnea and tumor pain). Only mild adverse reactions, primarily decreased appetite and fatigue, were observed. Furthermore, no severe grade III/IV adverse events were reported (97).

Despite the promise of FAP-targeted radionuclide therapy, its clinical deployment is limited by several factors: intra- and intertumoral heterogeneity of FAP expression; suboptimal pharmacokinetics for certain ligands; the limited quantity and quality of clinical evidence; and inadequate standardization of imaging and therapeutic workflows. Priorities for future work include: optimizing molecular design and chelation/radiolabeling strategies to enhance *in vivo* retention and tumor selectivity; enabling precise patient stratification and individualized dosing guided by quantitative FAP PET metrics; rigorously assessing combinations with chemotherapy, immunotherapy, and external-beam radiotherapy; and starting large-scale, prospective investigations with extended observation periods to drive standardized, scalable use across LC care (105).

### 4.2 SSTR2-targeted radionuclide therapy

Somatostatin receptor 2(SSTR2) is widely present across neuroendocrine neoplasms; in small-cell lung cancer (SCLC), about 50% of tumors express SSTR2 at varying levels, yet ligand uptake is generally lower than in other NETs. Moreover, commonly used SCLC cell lines (H446 and H69) exhibit low SSTR2 expression (106–108). Because SSTR expression is often heterogeneous across lesions within the same patient, comprehensive SSTR PET imaging should be performed before treatment selection to evaluate receptor expression in all relevant sites and determine eligibility for SSTR-targeted radionuclide therapy. An expanding clinical literature supports peptide receptor radionuclide therapy as a promising approach for lung neuroendocrine tumors. Contraindications to SSTR-targeted radionuclide therapy include absent SSTR expression in the setting of active disease sites, during pregnancy, with moderate–severe kidney, liver, or heart dysfunction, and in the presence of blood-related abnormalities (109). Accordingly, the anticipated benefits should be weighed against potential risks prior to therapy.

The principal SSTR2-targeted radionuclide therapies used in pulmonary neuroendocrine tumors (NETs) are [^177^Lu]Lu-DOTATATE (lutetium-177–labeled somatostatin analog) and [^90^Y]Y-DOTATOC (yttrium-90–labeled somatostatin analog). A 2024 systematic review encompassing 27 clinical studies of pulmonary NETs pooled outcomes from PRRT regimens containing ^177^Lu and/or ^90^Y and reported a combined objective response rate (ORR) of 25.6% (range, 5.3%–66.7%) (110). Notably, response to PRRT varies substantially across pulmonary NET subtypes. Two large retrospective cohorts underscored differences between pulmonary NETs and gastroenteropancreatic NETs (GEP NETs): one study reported a median PFS of 11 months for lung NETs, compared with 20–22 months for GEP NETs, with the second cohort showing a comparable pattern (111, 112). These disparities are likely influenced by multiple confounders, including tumor biological heterogeneity, differences in SSTR expression, and variability in treatment parameters (e.g., radionuclide, administered activity, and number of cycles).

In LC, Previous studies have confirmed that peptide receptor radionuclide therapy (PRRT) exhibits promising antitumor efficacy in mouse models bearing non-small cell lung cancer (NSCLC) xenografts. In addition, somatostatin analogs (SSTA) conjugated with chemotherapeutic agents such as doxorubicin (DOX) or radionuclides like ¹³¹I have also demonstrated therapeutic potential in tumor-bearing mouse models. Taking ¹^88^Re-labeled somatostatin analogs (SSA) as an example, a variety of radiolabeled peptide conjugates have entered preclinical or early-stage clinical evaluation. As early as 2013, a study systematically evaluated [¹^88^Re]Re-MAG3-depreotide and demonstrated that it effectively inhibited proliferation and invasion, induced apoptosis in non-small cell lung cancer (NSCLC) cell lines, and significantly suppressed tumor growth in a nude mouse xenograft model (113). Notably, this study further revealed the role of radiolabeled somatostatin analogs in inhibiting tumor angiogenesis, suggesting that their antitumor effects may be mediated through multiple mechanisms beyond direct cytotoxicity. These findings provided important experimental evidence for receptor-mediated radionuclide therapy in NSCLC. Subsequently, [¹^88^Re]Re-P2045, an innovative peptide derivative of somatostatin analogs, entered clinical trials in 2017. The phase I/II study (NCT02030184) was designed to systematically evaluate the therapeutic efficacy of this agent in small cell lung cancer and other advanced neuroendocrine neoplasms; however, the trial was prematurely terminated due to dose-limiting nephrotoxicity (114, 115). Collectively, these findings indicate that although ¹^88^Re-labeled somatostatin analogs hold potential value in the treatment of lung cancer, their clinical translation remains challenged by safety concerns related to nephrotoxicity. Future efforts should focus on optimizing ligand design and administration strategies to achieve a more favorable balance between efficacy and toxicity. Building on these findings, another study explored the potential of ^211^At-labeled octreotide ([^211^At]At-SPC-octreotide), an α-particle-emitting radiopharmaceutical, for targeted radionuclide therapy in NSCLC. In this study, a subcutaneous xenograft model was established using the human NSCLC cell line A549, and the antitumor effect was evaluated following intratumoral injection. Histopathological analysis revealed that ^211^At-labeled octreotide induced significant apoptosis in tumor cells in a dose-dependent manner (116). The unique advantages of α-particle therapy render it particularly suitable for the treatment of minimal residual disease and micro-metastases. This inherently defines the patient population most likely to benefit in future clinical trials—namely, those with a low burden of disseminated disease that is difficult to adequately cover with conventional β-emitters or external beam radiotherapy. Building on this precision-oriented rationale, the present study further provided preliminary biodistribution data, establishing an animal model foundation for subsequent human dose estimation. Concurrently, thyroid radioactivity uptake was monitored as a key indicator to indirectly assess off-target toxicity. The results demonstrated that thyroid radioactivity levels remained consistently low in the [^211^At]At-SPC-octreotide treatment group, in stark contrast to the free ²¹¹At group. These findings suggest that the targeted delivery strategy effectively mitigates the risk of thyroid accumulation associated with free ²¹¹At. In summary, this study, specifically focused on NSCLC, validated the therapeutic potential of SSTR-targeted α-radionuclide therapy in this malignancy.

In 2024, Hartmut Rauch et al. systematically evaluated a combination strategy for SCLC in the context of limited therapeutic options and modest efficacy of SSTR2-targeted therapy: PARP inhibitors (olaparib, rucaparib) plus peptide receptor radionuclide therapy (PRRT) with Lutetium-177 labeled to DOTA-TOC. *In vitro*, SCLC cell lines exhibited low SSTR2 expression, and the addition of PARP inhibition substantially potentiated PRRT, reflected by lower required PRRT activity and greater DNA damage. In murine models, the combination significantly suppressed tumor growth, prolonged survival, and did not induce evident acute toxicity. Importantly, fractionation did not yield additional benefit, suggesting that single high-dose administration may be pivotal for efficacy (108). The authors concluded that PARP inhibitors act as effective radiosensitizers, markedly enhancing SSTR2-targeted PRRT in SSTR2-low SCLC and providing robust preclinical support for clinical translation. Building on this work, in 2025, Fabrice N. Njotu and colleagues conducted a systematic assessment of the new SSTR−targeted radiopharmaceutical [^225^Ac]Ac−EBTATE in SCLC. Pharmacokinetic, biodistribution, dosimetric, and safety assessments demonstrated an approximately 40.27-hour blood clearance half-life with sustained tumor uptake. At 2×34 kBq, tolerability over 28 days was favorable without significant nephrotoxicity or pulmonary injury, whereas weight loss at 2×74 kBq indicated dose-limiting toxicity. Efficacy analyses showed that in the SSTR2-high NCI-H524 model, 2×30 kBq achieved an 80% complete response rate and 100% survival; in the SSTR2-low NCI-H727 model, survival benefits were dose-dependent (117). Collectively, these data support a promising targeted radiotherapeutic approach for extensive-stage SCLC and justify advancement to clinical trials.

## 5 Conclusions and future directions

A major challenge in precision diagnosis and therapy of lung cancer (LC) lies in the limitations of traditional histopathological biopsy. Although regarded as the gold standard, this approach is constrained by tumor heterogeneity, sampling errors, and the risk of complications associated with invasive procedures, which can be as high as 17% (118, 119). Consequently, it often fails to comprehensively capture the molecular profile of individual tumors. In contrast, radionuclide molecular imaging using PET or SPECT enables non-invasive, *in vivo* quantification of lung cancer-specific targets—such as FAP, SSTR2, and c-Met—and allows for dynamic monitoring of the tumor microenvironment (TME) (120). This capability provides real-time guidance for precision patient stratification and individualized treatment decisions. These technological advantages position radionuclide-based imaging and therapy as a promising frontier in the advancement of lung cancer management.

Despite the promising potential of radionuclide therapy in lung cancer, its clinical application is associated with dose-limiting toxicities, primarily affecting the kidneys, bone marrow, and off-target organs. Nephrotoxicity is the most frequently observed concern, as radiopharmaceuticals are predominantly cleared via the renal route. Currently, data on nephrotoxicity associated with PSMA-targeted radioligand therapy in the treatment of lung cancer remain limited. Drawing on evidence from prostate cancer, clinical studies investigating [¹^77^Lu]Lu-PSMA (including PSMA-617 and PSMA I&T) in patients with prostate cancer have demonstrated that this therapeutic approach does exert a certain impact on renal function. In one study involving 32 patients, 10% of individuals exhibited progression from grade 1–2 to grade 3 nephrotoxicity. In the larger-scale VISION trial, the incidence of grade ≥3 renal adverse events in the [¹^77^Lu]Lu-PSMA-617 arm was 3.4%. These safety data derived from prostate cancer studies provide an important reference for evaluating the potential renal risks of radionuclide therapy in the context of lung cancer (121). Hematological toxicity reflects the high radiosensitivity of the bone marrow; for instance, [¹^77^Lu]Lu-pentixather may induce leukopenia and thrombocytopenia (122). Off-target organ damage can often be anticipated based on the physiological distribution of the target. As an example, GRPR-targeting agents pose a potential risk of pancreatic toxicity due to high pancreatic uptake (123). To mitigate these toxicities in the context of lung cancer treatment, a multidimensional proactive strategy should be implemented. For renal protection, co-infusion of amino acids such as lysine and arginine can be employed to competitively inhibit tubular reabsorption of radiopharmaceuticals. With regard to dosing, administered activity should be individualized based on patient body weight, renal function, and dosimetry results to optimize therapeutic delivery. Furthermore, ligand optimization is equally critical. Priority should be given to the development of modified ligands with smaller molecular weight, higher affinity, and reduced off-target uptake—such as FAPI derivatives modified with albumin-binding moieties—paired with radionuclides of appropriate energy range (e.g., α, β, or Auger electrons). Such an approach aims to achieve an optimal balance between efficacy and safety.

In summary, radionuclide therapy is progressively expanding from well-established applications in prostate cancer to the management of lung cancer, demonstrating unique theranostic value in this highly heterogeneous malignancy. A range of novel radiopharmaceuticals targeting key lung cancer-associated biomarkers(such as FAP, SSTR2, c-Met, and PD-L1) are currently under preclinical or early-stage clinical evaluation. Future research directions in lung cancer should focus on three core areas: overcoming tumor heterogeneity, optimizing combination regimens, and advancing precision toxicity management. To address tumor heterogeneity, the development of bispecific or multivalent ligands holds promise for capturing the expression variability both within individual tumors and across distinct lesions. Regarding combination strategies, exploring the optimal sequencing and dosing of radionuclide therapy in conjunction with immune checkpoint inhibitors and chemotherapy is of particular importance, especially in patients with driver gene-negative lung cancer. Concurrently, precision toxicity management is essential and should integrate patient-specific dosimetry with normal organ protection strategies to maximize the therapeutic window. With the continued maturation of α-emitting and Auger electron-labeled radiopharmaceuticals, along with advances in ligand engineering that enhance safety profiles, radionuclide therapy is poised to occupy a central role in precision oncology for lung cancer, offering new avenues to improve outcomes for patients with this highly lethal disease.
